# Supplementary material for: Association between Body Mass Index and Hospital Outcomes for COVID-19 Patients: A Nationwide Study
Source: J Clin Med. 2023 Feb 17;12(4):1617. doi: 10.3390/jcm12041617 (PMC9967784; doi:10.3390/jcm12041617)
Supplement: Supplementary file 1 [file jcm-12-01617-s001.zip › jcm-2195324-supplementary.pdf]

Supplementary Table S1: International Classification of Diseases, 10th revision, Clinical Modification (ICD-10-CM) codes used in data extraction.

	**ICD-10 codes**
**Outcomes**	
COVID-19 Infection	U071, U49, U50, U85
Mechanical Ventilation	5A1935Z, 5A1945Z, 5A1955Z
Septic Shock	R6521
**Comorbidities**	
Lung Cancer	C34X*, C399
Congestive heart failure	I50X*, I110, I130
Chronic pulmonary disease	J40X*, J41X*, J42X*, J43X*, J44X*, J45X*, J47X*, J84X*
Smoking	F172X*, Z720, Z87891
Type 2 Diabetes Mellitus	E11X*
Chronic Kidney Disease	N18X*
**Body Mass Index (BMI) Categorizes**	
BMI<19	Z681
BMI 20-29.9	Z682X*
BMI 30-39.9	Z683X*
BMI 40-49.9	Z6841, Z6842
BMI>50	Z6843, Z6844, Z6845

*X represents all ICD-10-CM codes starting with the alphanumeric code proceeding the X
